# Formula for Extempore Gaseous Chalybeate Water

**Published:** 1846-08

**Authors:** 


*Formula for extempore gaseous Chalybeate Water.*—Take of crystallized sulphate of iron two drachms, white sugar three drachms; pulverize and divide into twelve powders. Take of bicarbonate of soda two drachms, sugar three drachms, and divide also into twelve powders. One of each is mixed in half a tumbler of water, and drunk while effervescing.—*Medical Gazette*, Sept. 12, 1845.

